# Critical analysis of descriptive microRNA data in the translational research on cardioprotection and cardiac repair: lost in the complexity of bioinformatics

**DOI:** 10.1007/s00395-025-01104-1

**Published:** 2025-04-09

**Authors:** Mariann Gyöngyösi, Julia Guthrie, Ena Hasimbegovic, Emilie Han, Martin Riesenhuber, Kevin Hamzaraj, Jutta Bergler-Klein, Denise Traxler, Maximilian Y. Emmert, Matthias Hackl, Sophia Derdak, Dominika Lukovic

**Affiliations:** 1 Division of Cardiology, Department of Internal Medicine II, Medical University of Vienna, Vienna, Austria; 2 Ludwig Boltzmann Institute for Rare and Undiagnosed Diseases, Zimmermannplatz 10, 1090 Vienna, Austria; 3 Department of Cardiothoracic and Vascular Surgery, Deutsches Herzzentrum der Charite (DHZC), Berlin, Germany; 4 TAmiRNA GmbH, 1110 Vienna, Austria; 5 Core Facilities, Medical University of Vienna, Vienna, Austria

**Keywords:** Translational research, MicroRNA, Bioinformatics, Cardioprotection, Cardiac regeneration

## Abstract

The unsuccessful translation of cardiac regeneration and cardioprotection from animal experiments to clinical applications in humans has raised the question of whether microRNA bioinformatics can narrow the gap between animal and human research outputs. We reviewed the literature for the period between 2000 and 2024 and found 178 microRNAs involved in cardioprotection and cardiac regeneration. On analyzing the orthologs and annotations, as well as downstream regulation, we observed species-specific differences in the diverse regulation of the microRNAs and related genes and transcriptomes, the influence of the experimental setting on the microRNA-guided biological responses, and database-specific bioinformatics results. We concluded that, in addition to reducing the number of in vivo experiments, following the 3R animal experiment rules, the bioinformatics approach allows the prediction of several currently unknown interactions between pathways, coding and non-coding genes, proteins, and downstream regulatory elements. However, a comprehensive analysis of the miRNA-mRNA-protein networks needs a profound bioinformatics and mathematical education and training to appropriately design an experimental study, select the right bioinformatics tool with programming language skills and understand and display the bioinformatics output of the results to translate the research data into clinical practice. In addition, using in-silico approaches, a risk of deviating from the in vivo processes exists, with adverse consequences on the translational research.

**Supplementary Information:**

The online version contains supplementary material available at 10.1007/s00395-025-01104-1.

## Key points

1. **Translational challenges**: translating animal research to humans is difficult due to genetic and evolutionary differences, especially in cardiac regeneration and cardioprotection.
2. **Species-specificity**: miRNAs are key regulators in cardiac repair, but species-specific variations limit their translational utility.
3. **Bioinformatics tools impaired by incomplete annotation**: advanced tools help uncover conserved pathways but are hindered by incomplete annotations and species differences.
4. **Data complexity**: large, inconsistent datasets and species-specific effects require careful filtering and advanced computational expertise**.**
5. **Bioinformatic literacy**: bioinformatics expertise is needed to access, analyze, and interpret big data and computational network approaches.

**Supplementary Information:**

The online version contains supplementary material available at 10.1007/s00395-025-01104-1.

## Introduction

The unsuccessful translation of cardiac regeneration and cardioprotection from animal experiments to clinical applications in humans has raised some essential questions. Which animal model is optimal for translational research? Which molecules (e.g., miRNAs) involved in regulatory mechanisms serve as obstacles in this translational process? Can bioinformatics analysis close the gap between human and animal research outputs? The successful translation of animal models of certain human diseases depends on several factors, including the right choice of animal species and carefully designed experiments with statistical power, randomization, and blinded analyses of the pre-clinical studies [25, 71, 95, 138, 206, 230, 239, 257]. For example, in vitro models are useful for testing drug toxicity [30], small animals are practical for knock-out models or performing a large number of experiments [282], and large animals are convenient for myocardial infarction or cardioprotection models [239]. However, comparative functional genomics studies have revealed an average 70% genomic similarity between humans and zebrafish [101], or 85% and 95–98% in small and large animals, respectively [170, 175, 280, 314], depending on the type of gene (coding vs. non-coding) or locus, organ developmental stage, tissue, and cell subset [62]. This genomic dissimilarity lies in both the coding domain and the regulatory regions.

Interestingly, phylogenetically primitive species (e.g., jellyfish, worms, salamander, axolotl, zebrafish) possess the largest specific organ regeneration capacities [29, 156, 184]. Comparative genomics has shed light on conserved miRNA orthologs that maintain consistent functions across species regardless of their regenerative capacity [62, 122, 175, 314]. However, large animals with similar hemodynamics as humans and the greatest genomic similarity have less proficiency in cardiac regeneration and cardioprotection similar to humans [256]. Therefore, regeneration capacity has not been conserved in the cardiovascular organs during evolution, though it was retained in some other human organs, such as the skin [278]. This evolutionary discrepancy highlights the critical role of molecular regulators enabling organ regeneration, including non-coding RNAs (ncRNAs).

ncRNAs represent approximately 97% of all RNAs [199, 240] and significantly contribute to regulation of the transcription of messenger RNAs (mRNAs) into protein. Several ncRNAs have been demonstrated to interact in cardioprotection and regeneration in zebrafish, rodent, or pig models. However, despite these insights, comparative genomic analyses have revealed that approximately 16–26% of protein-coding genes identified in the mouse or domestic pig and 25% of the zebrafish genome are not annotated in humans. A missing regulatory matrix element (e.g., annotated miRNA in animals but non-annotated in humans) in the ncRNA–mRNA networks not only leads to a non-comparable pathway and network between species, but also to inaccuracies in downstream regulatory pathways [121, 123, 203], contributing to the translational gap between animal models and humans.

Bioinformatics and novel in-silico target prediction programs, combined with interactive network theoretical approaches, allow deep insights into the modulatory and regulatory effects of miRNAs and mRNA networks involved in diverse cellular processes and disease mechanisms that play a role in cardiomyocyte renewal or the preservation of cardiac function [190]. These advances, including network computational tools and artificial intelligence workflows, have narrowed the translational gap between humans and model organisms, enabling more precise and effective extrapolation of research findings from laboratory studies to clinical applications.

We reviewed the current landscape of research into miRNAs involved in cardioprotection and cardiac regeneration, with a focus on understanding the translational challenges across species and identifying gaps in the effective translation of findings from model organisms to humans.

## Methods

### Data collection

To establish an up-to-date collection of miRNAs relevant to cardioprotection and cardiac regeneration, we conducted a comprehensive literature review for the period between 2000 January and 2024 December in the following electronic publication databases: PubMed, CrossRef, Web of Science, and Scopus. We used the following search criteria: 1) cardiac: cardioprotection, conditioning, regeneration, ischemic conditioning, pre-conditioning; 2) miRs, miRNA, microRNA; 3) species: rodent, mouse, mice, rat, zebrafish, pig, porcine, human, patient. For the search in orthologs between species and annotations, and to display the results, we used the freely available databases listed in Table 1.

**Table 1:** Bioinformatic websites used for the current analysis

Name of the Database	Leader, owner, publisher	Year of foundation	Last version	Website	Ref
Ensembl	R. Finn, A. Yates, UK	1999	V 115	https://mart.ensembl.org/index.html	[179]
HUMANCYC	R. Caspi, P.D. Karp, et al	2003	V 28.5	https://humancyc.org/HUMAN/	[124]
HGNC	HUGO Gene Nomenclature Committee (HGNC), European Bioinformatics Institute (EMBL-EBI), UK	1996	18.03.25	https://www.genenames.org/tools/hcop/#!/	[181]
GeneCards	Weizmann Institute of Science	1996	23.12.2024 V 5.23	https://www.genecards.org/	[241]
miRDB	Xiaowei Wang's lab at the Department of Pharmacology, University of Illinois at Chicago	2008	June, 2019, V 6.0	https://miRdb.org	[48]
miRBase	S. Griffiths-Jones, D and Z Fan, The University of Manchester, UK	2002	2022 V22.1	https://mirbase.org	[82, 130]
miRWalk	Medical Faculty Mannheim of the Heidelberg University	2018	Aug. 2024 V3	http://miRwalk.umm.uni-heidelberg.de	[242]
Bioinformatics & Evolutionary Genomics	VIB/Ugent, Bioinformatics & Evolutionary Genomics, Belgium	n.a	n.a	https://bioinformatics.psb.ugent.be/webtools/Venn/	n.a
The Pig RNA Atlas	Collaboration between BGI and the Human Protein Atlas	2022	V 1.0	https://www.rnaatlas.org	[123]
STRING	STRING CONSORTIUM	2002	26.07.2023 V 12	https://www.string-db.org	[249]
Cytoscape	Cytoscape Consortium, National Institute of General Medical Sciences (NIGMS)	2001	2025 V 3.10.3	https://cytoscape.org	[169, 224]
mirnaverse	Bioinformatics/Medical Informatics department at Bielefeld University	2024	CC BY 4.0	https://www.mirnaverse.de/	[88]
HumiR	Clinical Bioinformatics at Saarland University	2020	30.09.2024	https://ccb-web.cs.uni-saarland.de/humir/	[231]
TargetScan	Benjamin P. Lewis	2003	Sept. 2021; V8.0	https://www.targetscan.org/	[143, 144]
miRTarBase	ISBLab, School of MEDICINE and Warshel Institute for Computational Biology, The Chinese University of Hong Kong, Shenzhen, China	2011	2022; V 10.0	https://mirtarbase.cuhk.edu.cn	[106]
National Library of Medicine (NLM)	NLM | NIH | HHS | USA.gov	1968	2025	https://www.ncbi.nlm.nih.gov/gene/	[56]

References are selected based on the citation recommendations of the relevant websites

V: Version; Ref: Reference; n.a. data not available

The miRNAs relevant for cardiac regeneration and cardioprotection were divided into three types: 1) miRNAs with existing annotations in all investigated species (human, rat, mouse, pig, or zebrafish), 2) miRNAs with human, but different annotations between the species, and 3) miRNAs without an annotated function in humans but that were described in the literature as a result of next-generation sequencing or miRNA arrays. The miRNAs were tabulated with the available annotations and target mRNAs, extracted from the publications. Similarities between all species was displayed in a bar plot.

### Nomenclature

According to the accepted nomenclature, miRNA is commonly used for micro-RNA, whereas miR is commonly used as a prefix (e.g., miR- 1- 5p) [8]. We have separated the miRNAs with the same number but different suffix of − 5p or − 3p, recognizing that the − 3p and − 5p strand variants may target different mRNAs. In several early reports, the two strands of the pre-miRNA were differentiated based on their stability and function, with asterisk (*) labelling of the passenger strand, which is usually degraded. However, we have not retained the diverse letter or number suffixes for the same miRNAs (e.g., miRNA- 1a, miRNA- 1b, etc.) because these variants are either completely or almost identical in different species [8]. Additionally, some differences might exist between species in miRNA nomenclature even if sequence similarities are 100% (e.g. identical sequences of ssc-miR- 451, rno-miR- 451 - 5p and hsa-miR- 451a) [191].

### Functional comparison of miRNAs between species

To compare the molecular pathways showing an interaction between miRNAs and target genes between the species, cardiac regeneration or cardioprotection-relevant keywords were queried in the miRWalk Reactome database (http://mirwalk.umm.uni-heidelberg.de/genesets/) with online databases available for each species. Several pathways or gene sets were not listed for all species; for example, the cardiogenesis pathway was available only for humans (R-HSA- 9733709#Cardiogenesis). Therefore, we selected the myogenesis Reactome pathways, which are available for human, rat, and mouse (R-HSA- 525793#Myogenesis, R-MMU- 525793#Myogenesis and R-RNO- 525793#Myogenesis), listing all miRNAs involved in myogenesis in the three species.

### Comparison of downstream regulation of one miRNA in different species

To compare the downstream regulation of one miRNA with target genes, we selected miRNA- 375b, which plays a role in ischemia/reperfusion and is annotated in all species. We displayed the target gene similarities in humans, rats, and mice.

### Complexity of the human cardiogenesis miRNA network

To display the complete human cardiogenesis network, we downloaded the interacting miRNAs for all of the human protein-coding genes from the Reactome pathway (R-HSA- 9733709#Cardiogenesis) and displayed the miRNA-mRNA and inter-gene connections using Cytoscape (https://cytoscape.org) and Gephi (https://gephi.org) software. In addition, we selected three genes (*GATA4*, *MEFC2*, and *MYOCD*) and all their direct connections from the network to display in a separate subnetwork. We then chose the most cited miRNA (miRNA- 21, available for all investigated species) and displayed the miRNA- 21 network that interconnects with all regulated genes.

## Results

### Search results

**Fig. 1:**
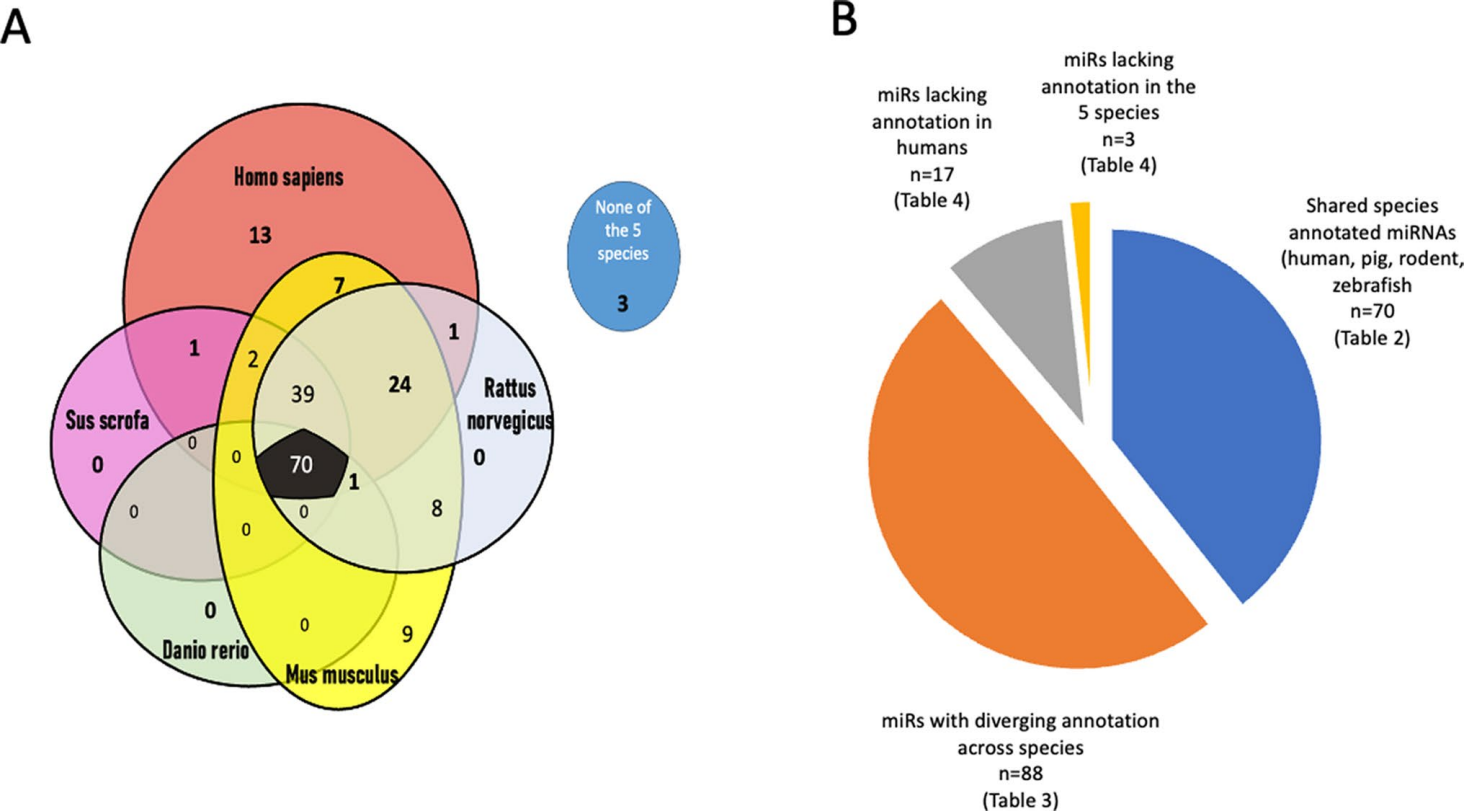
The miRNAs involved in cardiac protection and cardiac regeneration. We identified a total of 178 miRNAs involved in cardiac protection and cardiac regeneration in the literature search. **a** Venn diagram displaying the number of 175 common miRNAs in the five species (humans, pigs, rats, mouse and zebrafish), and three miRNAs without annotation in the investigated five animal species. **b** Summary of the miRNAs with common or diverse annotation in all five species

We identified 3464 papers matching the search criteria. After excluding reviews [13, 14, 49, 67, 81, 133, 178, 185, 186, 190, 196, 198, 212, 217, 229, 232, 237, 255, 256, 263, 265, 292, 336] and cell culture experiments, 332 remaining publications reported on miRNAs in cardioprotection and cardiac regeneration. We identified a total of 178 miRNAs that play a role in cardiac function preservation or improvement (Fig. 1). To list the species-specific miRNAs, we used the miRDB and National Library of Medicine (NIH) (https://www.ncbi.nlm.nih.gov/gene/).

Importantly, there are several miRNAs that do not have human orthologs, but have been analyzed in tissue development in other species (e.g., miRNA- 2 or miRNA- 13 or miRNA- 14) [26, 131, 193] or investigated in vitro. We excluded these papers from further analysis.

We found more than 190 miRNA databases, 120 of which are currently active [88].

### Variation in miRNA annotations across species and miRNA databases

Our analysis revealed significant variations in miRNA annotations across species, but also across the miRNA databases. Of the 178 miRNAs, 70 (39.3%) shared common annotations across humans, zebrafish, rodents, and pigs (Fig. 1). Table 2 presents the orthologous miRNAs among all investigated animals and humans, with the target genes if reported (date of extraction: 12.01.2025), whereas 88 (49.4%) miRNAs had different annotations among the species (Table 3, date of extraction: 12.01.2025). Table 4 lists the 20 miRNAs (11.2%, date of extraction: 12.01.2025) with a lack of annotation in humans but described as having a beneficial role in cardiac function (Fig. 1). Several of these miRNAs are not included in any miRNA-mRNA or miR-mRNA-protein networks (e.g., miRNA- 3107), hindering the integration of these miRNAs into functional networks. However, this does not exclude a role in specific transcriptomic processes. The number of miRNAs conserved between humans and pigs seems to be lower than the number conserved between humans and rats, mice, or zebrafish [7, 154, 201]. This could be due to pigs being a less frequently investigated species because of the high cost required to maintain the animals and the complexity of the experimental performance. Therefore, several databases, such as miRwalk (http://miRwalk.umm.uni-heidelberg.de), do not contain pig ncRNAs.

**Table 2:** List of miRNAs with functions in cardioprotection and regeneration, annotated in all 5 species (human/homo sapiens; prefix: hsa-/, pig (sus scrofa, prefix: ssc/, rat/rattus norvegicus, prefix: rno/, mice/mus musculus, prefix: mmu), zebrafish (danio rerio, prefix: dre), with targeted genes, if published

miRNA ID	Published miRNA variant	Published miRNA target genes	References
miRNA- 1	miRNA- 1, miRNA- 1- 3p	HSP60, HSP70, CASP9, Cx43, Amhc1, Tbx5 and Gata4, Mef2c, Cripto, CrabpII, Rarß, CrabpI, Hdac4, Calm1/Calmodulin, Erk2/Mapk1, DDR2, eNOS, iNOS, HSF- 1, PDCD4, Bcl- 2, Bax, Bid, Bcl10, Cidea, Ltbr, Trp53, Fas, Fasl, Bag3, Prdx2, IGF- 1, Bcl- 2, VE-cadherin, PECAM1, HSF- 1	[11, 21, 39, 52, 65, 78, 103, 115, 116, 168, 223, 238, 258, 268, 275, 309, 310]
miRNA- 7	miRNA- 7j miRNA- 7 k miRNA- 7a- 5p	Hspa1b,	[33]
miRNA- 9	miRNA- 9- 5p	Fstl1, SOCS5, SIRT1	[286, 318]
miRNA- 10	miRNA- 10a- 5p		[127]
miRNA- 15	miRNA- 15a miRNA- 15b miRNA- 15b- 5p miRNA- 15a- 5p		[35, 74, 111, 122, 168, 214]
miRNA- 16	miRNA- 16 miRNA- 16 - 5p	mTOR pathway	[35, 63, 71, 111, 168, 291]
miRNA- 17	miRNA- 17 miRNA- 17 - 3p miRNA- 17 - 5p	STAT3, Egln3/PHD3, AKT, mTORC2, TIMP- 3, PTEN, EGFR, JNK, SP- 1,	[44, 103, 168, 214, 219, 226]
miRNA- 20	miRNA- 20a miRNA- 20a- 5p miRNA- 20b- 5p	Egln3/PHD3, ULK1, ET- 1, TxA2, ANGII, PTEN, eNOS, PGI2, VEGF	[103, 168, 214, 219, 262, 331]
miRNA- 21	miRNA- 21 miRNA- 21 - 5p	PTEN, MMP- 2, SNHG1, PDCD4, SKP2, Cyt c, Bax, caspase- 3, HIF1α, Peli1, FasL, Per2, Akt, T1DM, eNOS, HSP70, HSF- 1	[6, 11, 14, 38, 39, 63, 65, 83, 103, 125, 172, 195, 202, 210, 214, 238, 254, 268, 293, 302, 309, 310, 335]
miRNA- 22	miRNA- 22	p38, MAPK, CBP, Jun-AP- 1, Jun-AP- 1, Bcl- 2, Bax, TNFalpha, IL- 6, Erα, Sp- 1, CREB, p21, p53	[63, 272, 304–306]
miRNA- 23	miRNA- 23a	Caspase- 7	[74, 75, 163, 176, 309]
miRNA- 24	miRNA- 24 miRNA- 24–1 - 5p miRNA- 24 - 3p	Cdip1, Tnfsf10, Bim, RIPK1, KEAP1, Nrf2, HO- 1, GATA2 and the p21-activated kinase PAK4, BAD (Bcl-XL/Bcl- 2-associated death promoter), Sirtuin1, eNOS, HSP70, HSF- 1	[11, 72, 100, 103, 120, 185, 208, 238, 247, 250, 268, 309, 310]
miRNA- 26	miRNA- 26 miRNA- 26a miRNA- 26b	PTGS2	[63, 238]
miRNA- 27	miRNA- 27a miRNA- 27b miRNA- 27a- 5p miRNA- 27b- 3p miRNA- 27b- 5p	Sik1, Ccnd2, Ccnd1, Cdc34, Cdk4, Cdk6, Ccne1, Ccnb1, CRTC1, CREB1, Gpx4	[63, 77, 195, 275, 285]
miRNA- 29	miRNA- 29a	Follistatin-like 1, JAK2/STAT3, Serca2a, Ryr2, S100a1, IGF- 1R, Apaf- 1, Hmox- 1, Cycs, Col-I, Col-III,	[11, 150, 180, 191, 296, 325]
miRNA- 30	miRNA- 30a miRNA- 30b miRNA- 30-d miRNA- 30e	CSE, H2S, p53, Map3k5, Map2k7, and Map2k4, Angpt14, Eif5a, Egr1, Irs2, Cebpb, Tsc22 d3, Dpep1	[36, 39, 63, 73, 127, 165, 225, 329]
miRNA- 31	miRNA- 31	PKCε, NFκB,	[195, 214, 243, 277]
miRNA- 34	miRNA- 34a miRNA- 34b miRNA- 34c	Acsl4, SIRT1, HNF4α, SOX2, CTCF, N-Myc, VEGFs, vinculin, protein O-fucosyltranferase 1, Notch1, PNUTS, sirtuin 1, Cyclin D1, Va	[168, 191, 195]
miRNA- 92	miRNA- 92b- 3p miRNA- 92–1 miRNA- 92–2	AKT, mTORC2, integrin subunit alpha5, MAP3 K2	[11, 23, 39, 97, 219, 266, 328]
miRNA- 93	miRNA- 93 - 5p	IL- 1β, IL- 18, caspase- 1, NLRP3, TXNIP, GSDMD, SOD, ROS,	[35]
miRNA- 99	miRNA- 99	FNTb, SMARCA5, CX43, MYL7	[4, 168]
miRNA- 100	miRNA- 100	FNTb, SMARCA5, CX43, MYL7	[4, 57, 168]
miRNA- 101	miRNA- 101a	DUSP1, MAPK p38, NF-κB pathways	[125, 289]
miRNA- 103	miRNA- 103	FADD, RIPK	[74, 118, 222, 315]
miRNA- 107	miRNA- 107		[63, 74, 118]
miRNA- 122	miRNA- 122 miRNA- 122 - 5p	SOCS1, CAT, SOD, GSH-px, GIT1, Sirt- 6/ACE2	[1, 52, 127, 222, 235, 320]
miRNA- 124	miRNA- 124		[92]
miRNA- 125	miRNA- 125b- 5p	Map3k5, Map2k7, and Map2k4, MTFP1, BRCA1, BIM, STAT2, PDE4a, CASQ1, NFAT5, XBP1, MAP3 K12, CPT2, FoxO1, MTRF1, TAZ	[60, 63, 151, 248, 257, 274, 275, 329]
miRNA- 126	miRNA- 126	HIF- 1α, PI3 K/AKT/eNOS and MAPK signalling pathway, AKT/GSK3β, VEGF,	[39, 54, 63, 79, 85, 93, 192, 221, 236, 283, 316]
miRNA- 128	miRNA- 128	Map3k5, Map2k7, Map2k4, p70 s6k1/p-p70 s6k1, Redd1, AKT, GSK3beta, Nrf2, TXNIP, Islet1	[37, 41, 74, 281, 329]
miRNA- 130	miRNA- 130 miRNA- 130a	PPAR-gamma	[51, 63, 103]
miRNA- 132	miRNA- 132		[12]
miRNA- 133	miRNA- 133 miRNA- 133b miRNA- 133a- 3p	MST1, STK4, TAOK1, mitoK-ATP, Ash1, VE-cadherin PECAM1, LTBP1, PPP2 CA, CASP9	[11, 28, 52, 70, 74, 91, 115, 118, 168, 227, 238, 275, 300]
miRNA- 135	miRNA- 135a- 5p	RasGAP-p120	[168]
miRNA- 138	miRNA- 138 - 5p	EGR1 TLR4 TRIF	[107, 107, 157, 181, 182]
miRNA- 139	miRNA- 139 - 5p	Stat1 pathway, BRCA1, BIM, STAT2, PDE4a, CASQ1, NFAT5, XBP1, MAP3 K12, CPT2, FoxO1, MTRF1, TAZ	[60, 127, 194, 258]
miRNA- 140	miRNA- 140 - 5p miRNA- 140 - 3p	Angpt14, Eif5a, Egr1, Irs2, Cebpb, Tsc22 d3, and Dpep1, NF-κB	[89, 92, 103, 165, 218, 243, 303]
miRNA- 141	miRNA- 141	ICAM- 1	[165]
miRNA- 142	miRNA- 142	FOXO1	[42, 127, 191, 214, 275]
miRNA- 143	miRNA- 143	Angpt14, Eif5a, Egr1, Irs2, Cebpb, Tsc22 d3, and Dpep1, Cepsilon, AMPK/Foxo1 pathway	[32, 42, 98, 165, 195]
miRNA- 144	miRNA- 144 miRNA- 144 - 5p	mTOR pathway, P-Akt, P-GSK3β, P-p44/42 MAPK, Zeb1/LOX1	[43, 92, 114, 137, 148, 275, 279]
miRNA- 145	miRNA- 145 miRNA- 145 - 5p	Binip3, Angpt14, Eif5a, Egr1, Irs2, Cebpb, Tsc22 d3, and Dpep1, LC3B, p62/SQSTM1,	[40, 63, 87, 96, 127, 165, 294, 330]
miRNA- 146	miRNA- 146a miRNA- 146 - 3p	Irak- 1, Traf6, NOX- 4, SMAD4, p65	[11, 12, 112, 183, 214, 221]
miRNA- 150	miRNA- 150	FOXD3-AS1, EGR2, P2X7R, CXCR4	[63, 114, 167, 214, 251, 332, 333]
miRNA- 155	miRNA- 155 - 5p	SOCS3, SIRT1, Cab39, AMPK, SHP2, ERK1/2	[99, 102, 109, 162, 222, 233]
miRNA- 181	miRNA- 181b- 5p miRNA- 181c	HAND	[81, 86, 168, 191, 258]
miRNA- 184	miRNA- 184		[197]
miRNA- 190	miRNA- 190a- 3p	CXCR4/CXCL12, GSH	[118]
miRNA- 192	miRNA- 192 - 5p	XIAP	[244, 258]
miRNA- 194	miRNA- 194 - 5p	RAC1	[147]
miRNA- 199	miRNA- 199a miRNA- 199b	TAOK1, Notch Ligand Jagged1, VEGF Signaling, p16, Rb1, Meis2	[47, 68, 142, 168, 191, 200, 214, 256, 327]
miRNA- 204	miRNA- 204a miRNA- 204 - 3p miRNA- 204 - 5p	Aff3, LGALS3, Jarid2	[158, 191, 213, 214, 287]
miRNA- 206		IGF- 1, TIMP3	[11, 39, 64, 118, 161, 223]
miRNA- 208	miRNA- 208a miRNA- 208a- 3p miRNA- 208 miRNA- 208b	MST1, STK4, TAOK1, LATS1, LATS2, VE-cadherin and PECAM1	[11, 27, 115, 118, 168, 228, 238, 243, 258, 298, 299]
miRNA- 210	miRNA- 210 miRNA- 210 - 3p	GPDH, ROS, Efna3 and Ptp1b, CDK- 10, β-catenin, Bcl- 2, APC, VEGF, p16, caspase- 3	[10, 12, 18, 74, 85, 104, 118, 141, 234]
miRNA- 212	miRNA- 212		[257]
miRNA- 214	miRNA- 214	HIF1 AN	[11, 214, 243, 260]
miRNA- 221	miRNA- 221	PUMA, ETS- 1, BNIP3, MAP1, LC3B, BBC3	[135, 138, 139, 213]
miRNA- 222	miRNA- 222	HIPK1, HMBOX1, PUMA, ETS- 1, T1DM, BNIP3, MAP1, LC3B, BBC3	[6, 136, 139, 140, 214]
miRNA- 223	miRNA- 223		[74, 118, 214, 316]
miRNA- 338	miRNA- 338		[164, 312]
miRNA- 345	miRNA- 345 - 5p	TLR4	[127, 145]
miRNA- 363	miRNA- 363	HAND	[86, 259]
miRNA- 375	miRNA- 375		[52, 59]
miRNA- 429	miRNA- 429	resistin	[295]
miRNA- 451	miRNA- 451 miRNA- 451a	PGC- 1α, mTOR pathway	[45, 74, 137, 166, 180, 191, 275]
miRNA- 491	miRNA- 491 - 5p		[103]
miRNA- 499	miRNA- 499 miRNA- 499 - 3p miRNA- 499a miRNA- 499 - 5p	MST1, STK4, TAOK1, TLR2, PKC, IL- 1β, IL- 6, PTEN, NF-κB, VE-cadherin and PECAM1	[11, 52, 67, 110, 115, 125, 127, 168, 214, 218, 266, 273, 308, 326, 337]
miRNA- 652	miRNA- 652		[258]
miRNA- 1825	MicroRNA- 1825	NDUFA10	[200]

Data extraction 15. January 2025 from the miRbase and https://www.ncbi.nlm.nih.gov/gene/ database

**Table 3:** List of miRNAs with functions in cardioprotection and regeneration, annotated in humans, with lack of annotation in some species

miRNA ID	Published miRNA variant	Human annotation with target gene, if applicable	Pig annotation with target gene, if applicable	Rat annotation with target gene, if applicable	Mouse annotation with target gene, if applicable	Zebrafish annotation with target gene, if applicable	References
miRNA- 18	miRNA- 18a- 5p	Yes	Yes	Yes	Yes	–	[168]
miRNA- 19	miRNA- 19 miRNA- 19a miRNA- 19b	Yes	–	–	Yes	–	[39, 103]
miRNA- 33	miRNA- 33a miRNA- 33	Yes	–	Yes	Yes	–	[103, 125]
miRNA- 98	miRNA- 98 miRNA- 98 - 5p	NGF, TRPV1	Yes	TLR4, PI3 K/Akt, DAPK1, TBX5, NGF, TRPV1	Yes	–	[33, 105, 245, 317, 323]
miRNA- 105	miRNA- 105–2	Yes	Yes	Yes	Yes	–	[191]
miRNA- 106	miRNA- 106 - 3p miRNA- 106 - 5p	Yes	–	Yes	Yes	–	[214]
miRNA- 127	miRNA- 127	Yes	Yes	Yes	Yes	–	[191]
miRNA- 148	mirNA- 148 - 3p	Yes	–	–	Yes	YES	[195]
miRNA- 149	miRNA- 149 - 5p	Yes	Yes	Yes	Yes	–	[33]
miRNA- 177	miRNA- 177	Yes	–	Yes	Hsp70.3	–	[253]
miRNA- 185	miRNA- 185 - 3p miRNA- 185 - 5p	CatK	Yes	Yes	Myd88	–	[33, 76, 146]
miRNA- 186	miRNA- 186 - 5p	LOX- 1	Yes	Yes	Yes	–	[61, 195]
miRNA- 188	miRNA- 188 - 3p miRNA- 188 - 5p	Yes	–	Yes	Yes	–	[258]
miRNA- 193	miRNA- 193a- 3p	Yes	Yes	Yes	Yes	–	[127, 191]
miRNA- 195	miRNA- 195 miRNA- 195 - 5p	Bcl- 2, PDCD4, T1DM	Yes	Yes	Yes	–	[6, 27, 103, 111, 168]
miRNA- 197	miRNA- 197	Yes	–	Yes	Yes	–	[316]
miRNA- 200	mirNA- 200a miRNA- 200a- 3p miRNA- 200b- 3p miRNA- 200c- 3p	Yes	–	Yes	Yes	Yes	[168, 275]
miRNA- 218	miRNA- 218 - 5p	Yes	Yes	Yes	Yes	–	[168]
miRNA- 294	miRNA- 294	Yes	–	Yes	Yes	–	[24, 275]
miRNA- 296	miRNA- 296 - 5p	Yes	Yes	Yes	Yes	–	[197]
miRNA- 297	miRNA- 297b- 3p	Yes	–		Yes	–	[275]
miRNA- 298	miRNA- 298 miRNA- 298 - 5p	Yes	–	BAX	Yes	–	[33, 288, 307, 324]
miRNA- 300	miRNA- 300 - 3p	Yes	–	Yes	Yes	–	[195]
miRNA- 301	miRNA- 301a	Yes	Yes	–	Yes	–	[103]
miRNA- 302	miRNA- 302 miRNA- 302 miRNA- 302b- 3p miRNA- 302c- 3p miRNA- 302 d- 3p	LATS1, LATS2, FOXO3, Mob1, Mst1, HSP20	–	–	Yes	–	[168, 173, 197, 211, 261]
miRNA- 320	miRNA- 320 - 5p	Yes	Yes	Yes	Yes	–	[69, 211, 258]
miRNA- 324	miRNA- 324 - 3p miRNA- 324 - 5p	Yes	Yes	BRCA1, BIM, STAT2, PDE4a, CASQ1, NFAT5, XBP1, MAP3 K12, CPT2, FoxO1, MTRF1, TAZ	Yes	–	[61, 103]
miRNA- 328	miRN- 328 miRNA- 328c- 5p	Yes	Yes	Yes	Yes	–	[33, 238, 258]
miRNA- 331	miRNA- 331 - 5p	Yes	Yes	Yes	Yes	–	[258]
miRNA- 335	miRNA- 335 - 3p miRNA- 335 - 5p	Yes	Yes	Yes	Yes	–	[33, 194, 258]
miRNA- 339	miRNA- 339	Yes	Yes	Yes	Yes	–	[191]
miRNA- 340	miRNA- 340 - 5p	Yes	Yes	Yes	Yes	–	[125]
miRNA- 346	miRNA- 346 miRNA- 346 - 3p	Yes	–	NFIB	CaMKIId	–	[290, 301, 334, 336]
miRNA- 361	miRNA- 361 miRNA- 361 - 5p	Yes	Yes	Yes	BAX, Cyt–C, PHB1	–	[270, 288, 321]
miRNA- 367	miRNA- 367 - 3p	Yes	–	–	HAND	–	[86, 168]
miRNA- 371	miRNA- 371a- 5p	Yes	–	–	–	–	[168]
miRNA- 373	miRNA- 373 - 3p	Yes	–	–	–	–	[168]
miRNA- 374	miRNA- 374a- 5p miRNA- 374b- 5p	Yes	Yes	Yes	Yes	–	[127]
miRNA- 377	miRNA- 377	Yes	Yes	Yes	Yes	–	[103]
miRNA- 378	miRNA- 378 miRNA- 378a- 5p	Yes	Yes	Yes	Ctgf, Hsp70.3	–	[126, 187, 195, 253, 258, 297, 322]
miRNA- 379	miRNA- 379 miRNA- 379 - 5p	TNFAIP8, CASP3 CASP9, NF-κB	–	Yes	Yes	–	[33, 53, 214, 218]
miRNA- 382	miRNA- 382 - 5p	Yes	Yes	Yes	Yes	–	[33]
miRNA- 384	miRNA- 384 miRNA- 384 - 3p	Yes	–	Beclin–1, HSP70	Yes	–	[108, 125, 319]
miRNA- 409	miRNA- 409 - 3p	Yes	–	Yes	Yes	–	[125]
miRNA- 410	miRNA- 410 - 3p	Fmr1	–	Yes	Yes	–	[152]
miRNA- 421	miRNA- 421	ACE2	Yes	Yes	Yes	–	[264]
miRNA- 423	miRNA- 423 - 3p miRNA- 423 - 5p	Yes	Yes	Yes	RAP2 C	–	[77, 171, 195]
miRNA- 424	miRNA- 424 - 3p miRNA- 424 - 5p	Yes	Yes	Yes	Yes	–	[127, 191]
miRNA- 431	miRNA- 431 miRNA- 431 - 5p	FBXO32	–	Yes	Yes	–	[153]
miRNA- 450	miRNA- 450a miRNA- 450b- 5p	-	SMAD2*	Yes	Yes	–	[33, 34, 191]
miRNA- 466	miRNA- 466b- 5p miRNA- 466a- 5p miRNA- 466i	Yes	–	Yes	Yes	–	[275]
miRNA- 467	miRNA- 467b	Yes	–	Yes	Yes	–	[275]
miRNA- 483	miRNA- 483 - 3p miRNA- 483 - 5p	Yes	–	Yes	VEZF1	–	[134, 309]
miRNA- 484	miRNA- 484	Yes	Yes	Yes	Yes	–	[33, 269]
miRNA- 486	miRNA- 486 - 5p	Yes	Yes	Yes	Mmp19, PTEN, FOXO1	–	[17, 33, 155]
miRNA- 487	miRNA- 487b miRNA- 487b- 3p	Yes	–	Yes	–	–	[168, 258]
miRNA- 494	miRNA- 494 miRNA- 494 - 3p miRNA- 494 - 5p	Yes	Yes	Yes	Yes	–	[74, 118, 258]
miRNA- 497	miRNA- 497 miRNA- 497 - 5p	Yes	Yes	IGF1, Smad7	Yes	–	[1, 46, 111, 119, 149, 243, 284]
miRNA- 500	miRNA- 500	Yes	Yes	Yes	Yes	–	[125]
miRNA- 503	miRNA- 503 - 5p	Yes	Yes	Yes	Yes	–	[258]
miRNA- 520	miRNA- 520a- 3p	UVRAG	–	–	–	–	[207]
miRNA- 522	miRNA- 522 - 3p	TNRC6 A	–	–	–	–	[117]
miRNA- 523	miRNA- 523	Yes	–	–	–	–	[33]
miRNA- 532	miRNA- 532 miRNA- 532 - 5p	ARC	Yes	Yes	caspase–3/7, prss23, EndMT	–	[16, 31, 33, 125, 258]
miRNA- 539	miRNA- 539 - 3p miRNA- 539 - 5p	ErbB4, AK003290	–	Yes	Yes	–	[168, 252, 267]
miRNA- 542	miRNA- 542 - 3p	Yes	Yes	Yes	ADAM9	–	[9, 33, 164]
miRNA- 544	miRNA- 544	Yes	Yes	Yes	WDR12	–	[311]
miRNA- 545	miRNA- 545 - 3p	Yes	Yes	Yes	Yes	–	[127]
miRNA- 574	miRNA- 574 - 5p	Yes	Yes	–	Yes	–	[275]
miRNA- 590	miRNA- 590	Yes	–	Yes	Yes	–	[32, 87, 142, 313]
miRNA- 615	miRNA- 615 - 5p	VEGF, VEGF-AKT/e -S, IGF2, RASSF2	Yes	Yes	VEGF, VEGF–AKT/e –S, IGF2, RASSF2	–	[5, 113]
miRNA- 636	miRNA- 636	Yes	–	–	–	–	[197]
miRNA- 657	miRNA- 657	Yes	–	–	–	–	[103]
miRNA- 665	miRNA- 665	MST1, STK4, TAOK1	–	–	Yes	Yes	[168]
miRNA- 675	miRNA- 675 - 5p	Yes	–	Yes	Yes	–	[275]
miRNA- 708	miRNA- 708 - 5p	ADAMT17	Yes	TnnT2 and NKX2.5	Yes	–	[58, 197, 209]
miRNA- 762	miRNA- 762	Yes	–	YES	YES	–	[275]
miRNA- 873	miRNA- 873 - 5p	Yes	–	XIAP	RIPK1/RIPK3, NRF	–	[246, 271]
miRNA- 877	miRNA- 877 - 5p	Yes	–	Yes	Yes	–	[258]
miRNA- 937	miRNA- 937 - 3p miRNA- 937 - 5p	Yes	–	–	–	–	[197]
miRNA- 1275	miRNA- 1275	Yes	Yes	–	–	–	[103]
miRNA- 1291	miRNA- 1291	Yes	–	–	Yes	–	[182]
miRNA- 1973	miRNA- 1973	Yes	–	–	–	–	[103]
miRNA- 3199	miRNA- 3199	Yes	–	–	–	–	[197]
miRNA- 4443	miRNA- 4443	Yes	–	–	–	–	[197]
miRNA- 4466	miRNA- 4466	Yes	–	–	–	–	[197]
miRNA- 4685	miRNA- 4685 - 5p	Yes	–	–	–	–	[197]
miRNA- 6815	miRNA- 6815 - 5p	Yes	–	–	–	–	[197]

Data extraction 15. January 2025 from the miRbase and https://www.ncbi.nlm.nih.gov/gene/ database

Yes means available annotation, but lack of target gene in the cited references

*miRNA- 450 complete list of target genes: SMAD2, VPS35, PPM1L, AP1M1, TNRC6 A, AAK1, DNAJC10, RNF169, RPS6 KA5, HS2ST1, CIITA, MTHFD2, GNL3L, RGMA, GPC6, ZBTB25, FAM78B, MED28, CBX5, EIF5 A2, DCTN5, KIAA1958, PDK3, ATE1, GULP1, GGCX, CEP57L1, TNPO1, DAPK2, DGKH, PAIP2B, APBB2, GPATCH2, NABP1, CPPED1, DBT, SNX19, CHN2, EXOSC2, SPRTN, RRM2B, CCDC50, NFIC, PAQR3, CHD9, ARHGEF18, SOD2, TOR1, AIP2, ORAI2, CADM1

**Table 4:** List of miRNAs with functions in cardioprotection and regeneration but lacking annotations in human and other species

miRNA ID	Published miRNA variant	Human annotation with target gene, if published	Pig annotation with target gene, if published	Rat annotation with target gene, if published	Mouse annotation with target gene, if published	Zebrafish annotation with target gene, if published	References
miRNA- 49	miRNA- 49	–	–	–	–	–	[111]
miRNA- 207	miRNA- 207	–	–	Yes	Yes	–	[214]
miRNA- 290	miRNA- 290 - 5p	–	–	Yes	Yes	–	[275]
miRNA322	miRNA- 322 - 5p	–	–	Yes	Yes	–	[33, 258]
miRNA- 327	miRNA- 327	–	–	Yes	Yes	–	[214]
miRNA351	miRNA- 351 - 5p	–	–	Yes	Yes	–	[33]
miRNA- 434	miRNA- 434 - 3p	–	–	Yes	Yes	–	[195]
miRNA- 551	miRNA- 551b	–	–	–	–	–	[275]
miRNA- 706	miRNA- 706	–	–	–	Yes	–	[275]
miRNA- 763	miRNA- 763	–	–	–	Yes	–	[275]
miRNA- 805	miRNA- 805	–	–	–	Yes	–	[275]
miRNA- 1192	miRNA- 1192	–	–	Yes	CASP3	–	[276]
miRNA- 1254	miRNA- 1254	–	–	–	–	–	[15, 55]
miRNA- 1954	miRNA- 1954	–	–	–	Ppp1r16b	–	[287]
miRNA- 1983	miRNA- 1983	–	–	–	Yes	–	[33]
miRNA- 3107	miRNA- 3107	–	–	–	Yes	–	[33]
miRNA- 3559	miRNA- 3559	–	–	Yes	Yes	–	[125]
miRNA- 3562	miRNA- 3562	–	–	–	Yes	–	[125]
miRNA- 6236	miRNA- 6236	–	–	–	Yes	–	[33]
miRNA- 6240	miRNA- 6240	–	–	–	Yes	–	[33]

Data extraction 15. January 2025 from the miRbase and https://www.ncbi.nlm.nih.gov/gene/ database

Yes means available annotation, but lack of target gene in the cited references

Notably, there are some discrepancies in the annotations and nomenclature between the different databases, most probably due to different database update times. For example, miRNA- 522 has a human annotation in miRDB but not in GenCYC, and miRNA- 523 has a human annotation in MiRGeneDB but not in HGNC or HumanCYC (Table 3).

### Different miRNAs for the same biological function in humans, rats, and mice

**Fig. 2:**
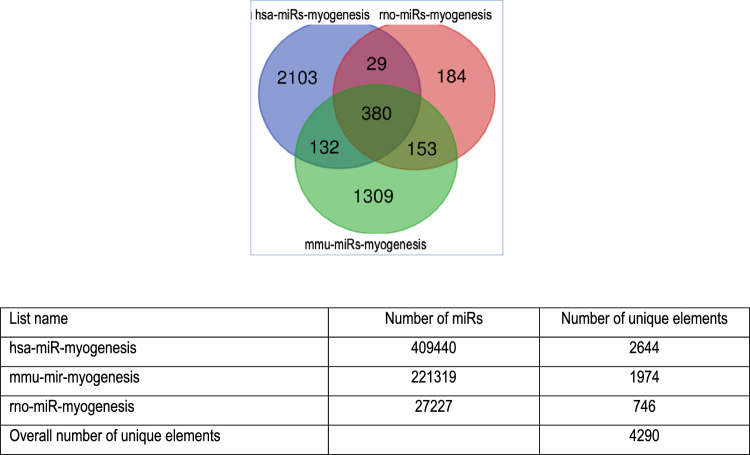
Venn diagram of miRNAs with a biological function in myogenesis in humans (hsa-miRNAs), rats (rno-miRNAs), and mice (mmu-miRNAs). Among the 4290 miRNAs, only 380 were common in all species. The miRNAs were retrieved from the Reactome pathways for myogenesis from http://miRwalk.umm.uni-heidelberg.de (R-HSA- 525793#Myogenesis, R-MMU- 525793#Myogenesis and R-RNO- 525793#Myogenesis) and displayed using https://bioinformatics.psb.ugent.be/webtools/Venn/ (date of data extraction: 17.02.2024)

When selecting the Reactome myogenesis pathways of rats, mice, and humans, different miRNAs are apparently responsible for myogenesis (Fig. 2). For example, miRNA- 28c is only in mice, miRNA- 7b is only in rats, and miRNA- 107 is only in humans. Among the 4290 miRNAs playing a role in myogenesis, only 380 (8.8%) are annotated commonly in humans, rats, and mice.

### Diverging downstream effects of the same miRNAs in different species

**Fig. 3:**
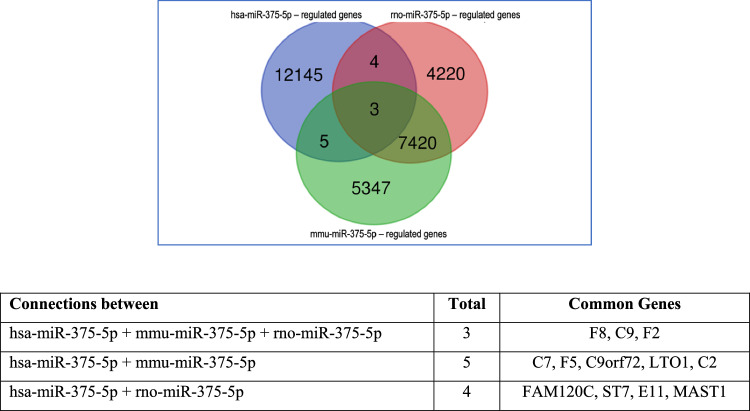
Venn diagram of miR- 375 - 5p—mRNA downstream regulation in humans (hsa-miR- 375 - 5p), rats (rno-miR- 375 - 5p), and mice (mmu-miR- 375 - 5p). All miR- 375-targeted genes are included. Only three genes are common in all three species. The miR- 375 - 5p downstream-regulated genes were retrieved from http://miRwalk.umm.uni-heidelberg.de (date retrieved: 22.04.2024) and displayed using https://bioinformatics.psb.ugent.be/webtools/Venn/

Despite the phylogenic conservation of thousands of miRNAs, the downstream regulatory effect can vary markedly between species and organs [135]. Figure 3 shows an example of the differing downstream miRNA-mRNA regulation of miRNA- 375, which has been investigated in cardiac regeneration and cardioprotection in different species [59, 229]. Among 29,144 target mRNAs, only three target genes (F8, C9, F2) are commonly regulated between humans and rodents; these genes belong to the coagulation and complement pathways and are only marginally associated with cardiomyocyte function. Although 7420 genes are common in rats and mice, only four are commonly targeted by miR- 375 in humans and rats and five in humans and mice.

### Heterogeneity and database-specificity of bioinformatics results

Interestingly, only the miRNA-mRNA cardiogenesis network for humans was found in the Reactome database, but the cardiogenesis protein–protein network was found for all species and displayed in the String network database. The protein–protein network of cardiogenesis genes (retrieved from https://www.string-db.org database) revealed several similar genes among humans, rats, mice, and pigs, but the number of genes and connections between the genes are different, with some of the genes having no annotations in humans (Supplementary Fig. S1).

### Complexity of available data

**Fig. 4:**
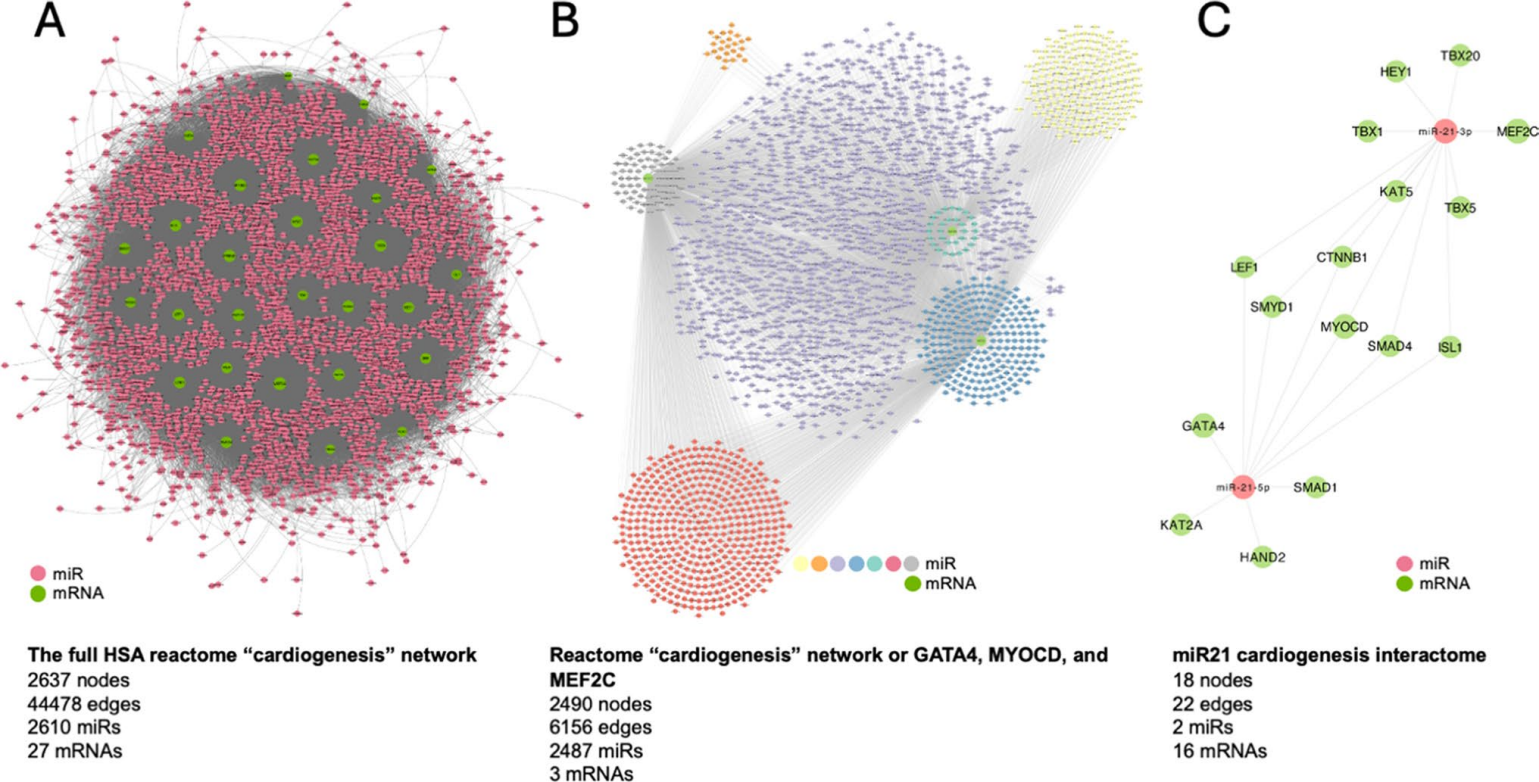
The miR-mRNA network of human cardiogenesis. Data were retrieved from the miRwalk Reactome network (date: 23.12.2024). **a** A total of 44,478 miR-mRNA connections among 27 genes and 2610 miRNAs with overlapping views of all connections. Network visualization by Gephi (https://gephi.org), Force Atlas (repulsion strength 200). **b** Focusing on three genes (*GATA4*, *MEFC2*, and *MYOCD*) and their direct connections forms a network of 2490 nodes (2487 miRNAs) and 6156 edges. Network visualization by Cytoscape (https://cytoscape.org), YFiles Organic Layout. **c** All interaction partners and interactions of miR- 21, forming a small network of 18 nodes and 22 edges. Network visualization by Cytoscape, YFiles Organic Layout. In all panels, mRNAs are green and miRNAs are salmon-colored

To fully understand the effect of miRNAs on the cardioprotection and cardiac regeneration processes, we have to add the appropriate molecular context. However, the addition of all putative miRNAs with their regulated mRNAs leads to much more complex and convoluted molecular interactions. For example, the human cardiogenesis miRNA-mRNA network (retrieved from the miRwalk Reactome network, date 23.12.2024) shows 197,296 miRNA-mRNA interactions, including 44,478 unique connections between 2610 miRNAs and 27 mRNAs (Fig. 4). This network, which is impossible to read with the naked eye, can only be analyzed using further computational filtering to identify the key players. Such filtering can be achieved using network properties of the miRNAs/mRNA themselves, such as centrality or connectivity. Additional data bioinformatics methods, such as using FDR or p-values, allow genes of interest to be selected or a focus on specific genes/miRNAs/pathways (Fig. 4). Thus, the exact filtering steps taken greatly influence the results at the end of the analysis.

### Experimental setting-derived regulation of cardioprotective genes by miRNA–mRNA networks

The expression of a gene may be different in different experimental settings or conditions, or even species-specific [100, 120, 250, 316]. For example, the downstream miRNA–mRNA regulation of miRNA- 24 - 3p has been investigated extensively in ischemia/reperfusion models in rats, mice, and humans. Interestingly, miRNA- 24 - 3p proved to be cardioprotective in in vivo ischemia/reperfusion models in rats [120] and mice [250], but the opposite effect was reported in mice, with blockade of miRNA- 24 decreasing the size of the myocardial infarction [72]. In contrast, in an ischemic limb experiment in mice, inhibition of miRNA- 24 - 3p led to an increase in dysfunctional microvessels, which impaired limb perfusion [177]. In temporary hind limb ischemia in humans, among other miRNAs, miRNA- 24 was increased at 1 h and remained elevated until 7 days after ischemia/reperfusion [316].

## Discussion

With this review and analysis, we aimed to shed light on the challenges researchers face in the heterogeneous landscape of miRNAs in cardioprotection and cardiac regeneration. Our review of the miRNA landscape revealed 178 miRNAs involved in cardioprotection and cardiac regeneration. By listing the miRNAs that play a role in preservation or improvement of cardiac function or prevention of ischemia/reperfusion injury, we were confronted with myriad data and non-comparable annotations of miRNAs in diverse databases, impeding a clear overview of the field.

Based on our findings, the original question of whether the bioinformatics analysis can close the gap between human and animal data cannot really be answered. In general, the successful translation of animal data to humans depends on multitudinous factors, including mechanistic factors identified in carefully designed pre-clinical studies with power calculations and blinded assessment of results in core laboratories; biological factors in vitro or in vivo in the choice of animals with human-like comorbidities and therapies; or molecular, genetic, or phenotyping factors among other genome similarities or translatomes with relevant -omics, single cell or single nuclei analyses, or other bioinformatics tools [25, 71, 95, 138, 229].

For example, monogenic modifications of inbred animal strains do not result in the complex situation of a heterogeneous, polygenic determined primordial risk constellation as seen in humans. However, there are some animal models with a polygenic background that may reflect the human situation better [66, 94, 128, 159, 160]. Nonetheless, analogous genotype does not guarantee phenotypic similarities [189].

In addition to high evolutionary conservation, many reports have described high turnover rate of certain miRNAs shared in different organisms, leading to phenotypic divergence [205], which may partially explain the inter-species differences. Chaudhari et al. performed a genome-wide investigation of the miRNA regulatory network in murine heart development up to 23 days post-birth and revealed fundamental roles of several miRNAs in the timely regeneration process [33]. Although all presented miRNAs are necessary for the Gene Ontology (GO) cellular processes and KEGG pathways, several of these miRNAs have no human annotation (Supplementary Fig. S2), limiting the translational applicability. This experiment is especially interesting because resection or cryoinjury of the heart apex of newborn mice (up to 7 days post-birth) leads to a robust cardiac regeneration with complete, or at least partially complete, restoration of the destroyed mammalian heart structure [90, 174, 204].

During acute pathophysiological conditions, such as ischemia or cell structure disorientation, the rapid dynamic regulation of miRNAs and the transcriptome leads to phenotypic fluctuation, influencing the cell’s biological function. Although bioinformatics tools offer the ability to take snapshots (e.g., single timepoint of tissue sampling) of tissue gene expression, it falls short of capturing the dynamic changes in biological processes in vivo and the 4-dimensional complexity of in vivo biologics. Neither bioinformatics nor in-silico research can simulate the myriad changes occurring in milliseconds in an organism, such as the ever-changing counter-regulation of blood pressure by heart rate or other actual neurohormonal conditions.

**Fig. 5:**
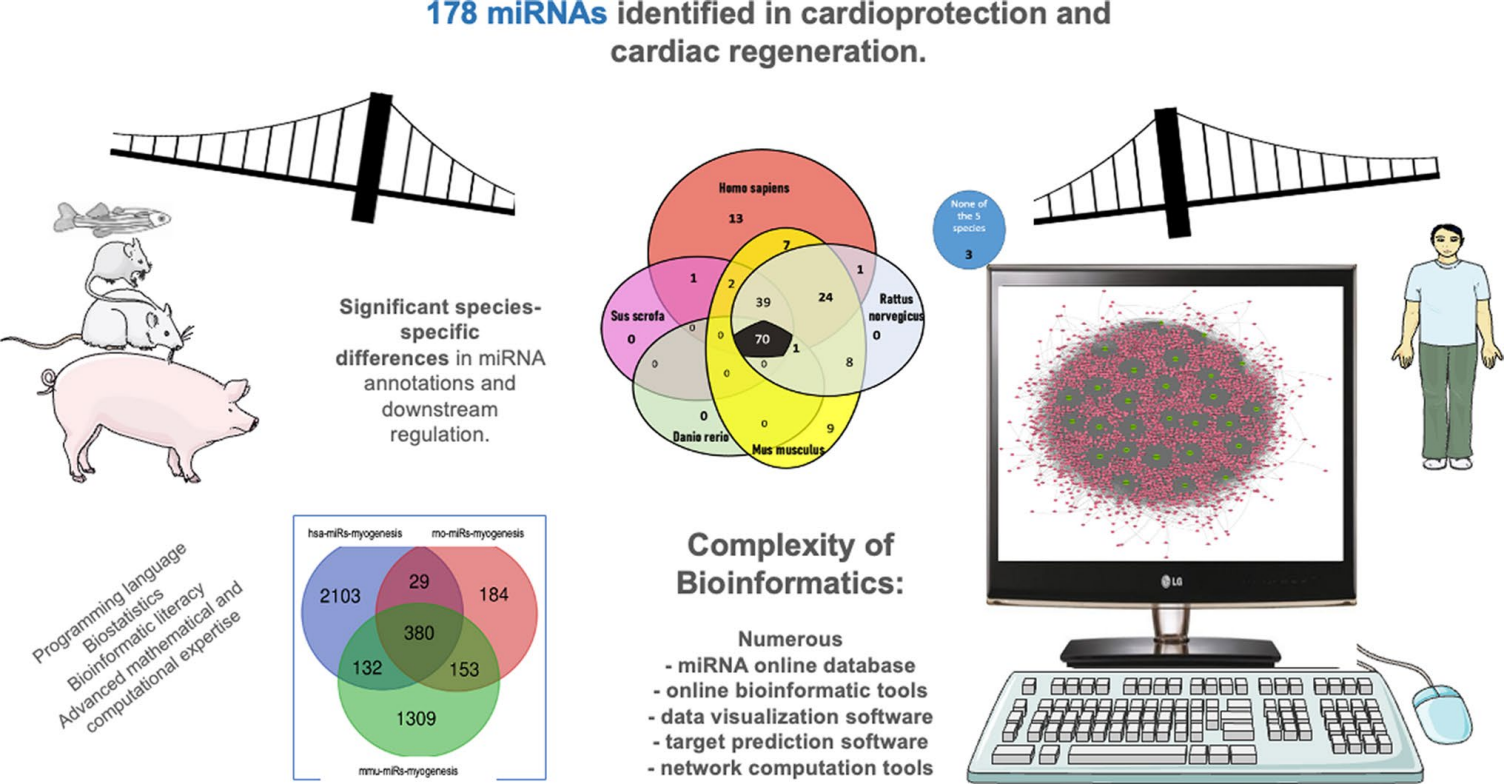
Graphical abstract

For the final downstream analysis of the miRNA-mRNA-protein networks, a physician or molecular biologist researching the topic of cardioprotection and cardiac regeneration needs a profound bioinformatics and mathematical education and training to appropriately design an experimental study; plan the analysis, including bioinformatics; select the right bioinformatics tool with programming language skills; and understand and display the bioinformatics output of the results to translate the research data into clinical practice. Thus, the analysis of miRNA-related data requires considerable computational literacy. Due to the vast amount of data available (as illustrated by the networks in Fig. 4), certain types of data analysis and filtering are required to work with these data. This filtering and analysis may be influenced by *p* values or other mathematical (e.g., false discovery rate, or pathway enrichment analysis, or strength score) or biological factors (e.g., correlation with circulating or myocardial tissue biomarkers, or statistical differences between the investigated groups, or other research targets specified by the research question), leaving the researcher to their own decision regarding what type of data, pathway database, expression dataset, data visualization tools, target prediction matrix, or network theoretical computations they should use, which affects the results of the analysis [2, 3, 19, 20, 22, 80, 84, 129, 132, 188, 215, 220]. In addition, a lack of sufficiently curated bioinformatics tools for specific research questions may lead to false assumptions. Furthermore, virtual genomics or proteomics results can be created by in-silico bioinformatics processes, and theories might be confirmed or discarded experimentally later. As a bridge between routine daily clinical practice in humans and computational biology does not (yet) exist, many clinicians trying to understand the translational multiplayer molecular networks with big data and artificial intelligence approaches are lost in the incomparable species-specific data and the complexity of bioinformatic approaches. Beyond the ultimate benefit of bioinformatics realizing the 3R animal rules (replacement, reduction, refinement), it might lead to a new level of translational failure (Fig. 5).

## Limitations

We conducted a pathway search using several key words, such as cardiac regeneration and cardioprotection, in several databases (KEGG, Pathbank, Signaling pathways, Biocarta, Spike, WikiPathways) but found no relevant pathway. Importantly, Chowdhury et al. reported considerable heterogeneity in pathway data, genetic and molecular networks, annotations, and updates and diversity in online automatic image reconstruction approaches [50]. We did not intend to create a new pathway or network, just to display the complexity of the miRNA databases and different downstream regulation in diverse species using available data and miRNA networks. As ncRNAs are 97% of the genome and miRNAs are the most phylogenetically conserved small regulatory RNAs, the cardioregenerative and cardioprotective functions of which have been thoroughly investigated, we chose the miRNAs with regulatory functions. Differences in signaling cascades among species and cardioprotective interventions have been described on the protein-level for years [216]. Therefore, it is not surprising that such differences may also be seen on the level of ncRNAs. Physiological or pathophysiological cardiac function depends on changes in the individual metabolism of free fatty acids and glucose, which differ between developmental biologies and species. As each miRNA affects hundreds and thousands of mRNAs and protein interactions, one change in miRNAs influences the downstream regulation of thousands of target mRNAs and proteins. Given that miRNAs represent less than 50% of ncRNAs, the involvement of other ncRNAs, such as lncRNAs or circRNAs, may also be critical in cardioprotection and cardiac regeneration, introducing additional complexity to translational research. Including further bioinformatics outputs with transcriptome, proteome, or metabolome analyses would be of interest, resulting in greater complexity of the translational processes but unnecessarily extending the length of this review.

There are several overlapping types of cardioprotection and cardiac regeneration, such as prevention of irreversible injury or cell death, inducing intrinsic (endogenous) regeneration, or pharmacological protection of the heart. We did not list the roles of miRNAs separately in these conditions. However, the main required outcome of all cardiac protective mechanisms is to reduce infarct size and microvascular obstruction, assuming at least partially similar mechanisms.

In conclusion, bioinformatics allows the prediction of several currently unknown interactions between pathways, coding and non-coding genes, proteins, and downstream regulatory elements. The ultimate benefit of this approach is a reduction of in vivo experiments, following the 3R animal experiment rules (replacement, reduction, refinement). On the other hand, such analysis may carry the risk of deviating from the in vivo processes, with adverse consequences on the translational research outputs. Our deep insight into the roles of miRNA in cardioprotection and regeneration explored an enormous amount of data, but the accession, analysis, and interpretation of these data requires considerable computational expertise, as well as a strong hypothesis to bridge the translational gap.

## Supplementary Information

Below is the link to the electronic supplementary material.

Supplementary file 1 (DOCX 1289 kb)
