# Supplementary material for: Critical analysis of descriptive microRNA data in the translational research on cardioprotection and cardiac repair: lost in the complexity of bioinformatics
Source: Basic Res Cardiol. 2025 Apr 9;120(3):443–72. doi: 10.1007/s00395-025-01104-1 (PMC12159128; doi:10.1007/s00395-025-01104-1)
Supplement: Supplementary file 1 — (DOCX 1289 kb) [file 395_2025_1104_MOESM1_ESM.docx]

**Supplementary file**

**Supplementary Fig. S1** Cardiogenesis gene networks in humans, mice, rats and pigs. Different gene networks, with mostly similar genes in the four species, are involved in cardiogenesis. The numbers of genes and connections between genes are different, and there are also several genes in rats and pigs that are not annotated in humans (red arrows). The cardiogenesis network pathway was retrieved from the String database (date: 22.04.2024) and displayed using the String database tool.

**Supplementary Fig. S2** Example of species differences during postnatal heart development. The miRNA regulatory network in mouse heart development up to 23 days post-birth. Several of these miRNAs have no human annotation (red). Increasing (yellow) and decreasing (blue) expression is shown with time. Adapted from Chaudhari et al. [19]
